# Random Stiffness Tensor of Particulate Composites with Hyper-Elastic Matrix and Imperfect Interface

**DOI:** 10.3390/ma14216676

**Published:** 2021-11-05

**Authors:** Damian Sokołowski, Marcin Kamiński

**Affiliations:** Faculty of Civil Engineering, Architecture and Environmental Engineering, Łódź University of Technology, Al. Politechniki 6, 90-924 Łódź, Poland; sokolowski.dmn@gmail.com

**Keywords:** particulate composites, hyper-elasticity, stochastic perturbation technique, probabilistic, homogenization method, stiffness tensor

## Abstract

The main aim of this study is determination of the basic probabilistic characteristics of the effective stiffness for inelastic particulate composites with spherical reinforcement and an uncertain Gaussian volume fraction of the interphase defects. This is determined using a homogenization method with a cubic single-particle representative volume element (RVE) of such a composite and the finite element method solution. A reinforcing particle is spherical, located centrally in the RVE, surrounded by the thin interphase of constant thickness, and remains in an elastic reversible regime opposite to the matrix, which is hyper-elastic. The interphase defects are represented as semi-spherical voids, which are placed on the outer surface of this particle. The interphase is modeled as hyper-elastic and isotropic, whose effective stiffness is calculated by the spatial averaging of hyper-elastic parameters of the matrix and of the defects. A constitutive relation of the matrix is recovered experimentally by its uniaxial stretch. The 3D homogenization problem solution is based upon a numerical determination of strain energy density in the given RVE under specific uniaxial and biaxial stretches as well as under shear deformations. The analytical relation of the effective composite stiffness to the input uncertain parameter is recovered via the response function method, using a polynomial basis and an optimized order. Probabilistic calculations are completed using three concurrent approaches, namely the iterative stochastic finite element method (SFEM), Monte Carlo simulation and by the semi-analytical method. Previous papers consider the composite fully elastic, which limits the applicability of the resulting effective stiffness tensor computed therein. The current study voids this assumption and defines the composite as fully hyper-elastic, thus extending applicability of this tensor to strains up to 0.25. The most important research finding is that (1) the effective stiffness tensor is sensitive to random interface defects in its hyper-elastic range, (2) its resulting randomness is not close to Gaussian, (3) the semi-analytical method is not perfectly suited to stochastic calculations in this region of strains, as opposed to the linear elastic region, and (4) that the increase in random dispersion of defects volume fraction has a much higher effect on the stochastic characteristics of this stiffness tensor than fluctuation of the strain.

## 1. Introduction

Multiscale computational methods and simulations have attracted scientists and engineers for many years, which has been documented by the comprehensive review presented in [1]. As is expected, such multiscale approaches are frequently connected with averaging and also with the homogenization method [2,3], where these two last techniques serve for remarkable reduction of computational complexity and material heterogeneity on a micro- or nano-scale. As it is known, some specific model reduction techniques [4] have also been concurrently resolved to minimize computer effort and preserve numerical accuracy.

Such multiscale homogenization methods have been worked out in solid mechanics to predict the elastic effective properties of fiber-reinforced composites [5], metal–ceramic composites with lamellar morphology [6] and polymeric agglomerated nanocomposites [7]. They have also used isogeometric FEM analysis to simulate thermomechanical contact at the composite interfaces [8]. The homogenization method in the context of multiscale modelling is employed in fluid mechanics, to model transport phenomena in some human tissues [9], for thermal transport simulation in polymer nanocomposites [10], to model electromagnetic and elastic couplings in CFRP composites [11], and finally to simultaneously carry out molecular simulations and multiscale homogenization in seepage and diffusion problems [12]. 

Nevertheless, the problem of stochastic micro or nanostructure and the realistic impact of the random spaces on the overall characteristics of the composite material (or system) is rather infrequent, and this is why it shall be discussed in this paper.

The linear mechanical behavior of single phase isotropic and anisotropic materials is well understood and has been proved by a firm theoretical background. This is also true for composites with non-complex internal composition and geometry, for which analytical solutions apply, and when geometry of the microstructure represents a certain pattern and symmetries. This allows them to be included in certain class of behaviors, e.g., cubic or orthotropic, etc. When such symmetries are not recognizable straight away, various homogenization approaches are used that usually require multi-scale considerations; some modern ones include the FE2 scheme [13] or neural networks [14]. In such cases, the representative volume element is selected to perfectly reflect the complex material microstructure and, thanks to the application of the homogenization method, to determine its effective properties whilst using a relatively small computational effort. Theoretical and numerical error bounds for macroscopic material characteristics can be estimated even before the final homogenization [15], but they may be very wide, especially for a larger contrast of the constituents’ properties. Composites after homogenization are assumed to be elastic and isotropic solids, but such an approach is perfect for small strain engineering applications and returns remarkable modelling errors in the case of incremental or dynamic loading and for the irregular microstructures. This has been illustrated in [16], where the influence of microstructure on the deformation process of particulate composites has been studied. This influence results from the fact that mechanical properties of composites in their original configuration before homogenization have a certain degree of anisotropy, which could be quantified by various measures, such as the Zener coefficient [17], universal anisotropy index [18] or tensor anisotropy index [19]. Anisotropy comes directly from the accidental microstructure and is present in all the composite stiffness tensor components calculated with the use of a series of specific boundary conditions. The initial assumption that this anisotropy will vanish together with an increase in the RVE may not be fully justified, even for particulate composites, and clearly depends on the manufacturing process as well as the loading conditions during the exploitation of a certain composite. This is why a convenient generation of composite microstructure is of paramount importance and needs the application of various mesh generation algorithms [20]. This is also a reason for the common usage of random geometry [21], where multiple realizations with a large set of arbitrary internal structures are used to determine a representative strength, stiffness or other, not only mechanical, properties [22,23]. Such studies, where randomness serves for a specific intermediate step, are only able to return some mean material properties or, at least, upper and lower bounds of these properties. When more information is required, the only possibility is a stochastic or probabilistic approach, which allows for the determination of further characteristics, such as the expected values, statistical dispersion or even the full probability densities of these macroscopic properties; some examples are demonstrated in [24,25]. A major problem in such considerations is a proper determination of the nature and properties of the input probability densities. This is because they require a very high number of repeated tests for identification of each random parameter; it is especially difficult for multi-scale analyses, where each scale has its own inputs. Sensitivity coefficients aid in solving this problem, because they determine the impact of all parameters on the macroscopic properties prior to the main analysis; only a few of the most influential input variables are sorted out to the final stochastic phase of computations. When statistical data are scarce, the most frequent choice of the probability density function (PDF) is the Gaussian one. Some studies based on other non-Gaussian inputs are also available, as in [26] for example. They generally require a more complex stochastic finite element method implementation and correspond to a situation, when statistical dispersion is not precisely known, but is bound to some specific interval only. 

Mechanical behavior in the non-linear regime of materials is already complex in the isotropic case, where multiple constitutive laws may be utilized for the same material. This is true for the hyper-elastic regime for instance, where these laws are usually defined assuming material isotropy, so that the potential application area is strictly limited. There also exists some hyper-elastic theories accounting for the anisotropy of material microstructures [27], however they are aimed at the representation of cartilage, arterial, brain or other biological tissues. Homogenization based studies in this specific nonlinear regime usually concentrate on a certain hyper-elasticity potential, for example the Ogden [28], Neo-Hookean [29] or van der Waals model [30]; some of them also compare the results of several potentials, as in [31]. Computation of random macroscopic properties in this regime becomes much more challenging than in the reversible elastic case, not only because of the lack of generality of the various laws but also from the computational view. 

Independently from the composites’ response under the given loads, one of the most important aspects of both deterministic and stochastic numerical modelling is the connection of their constituents. A perfect bonding is not achieved in many cases because of the chemical, thermal, electrical or mechanical properties’ incompatibilities and also due to manufacturing processes. In such cases, an interphase is inserted in-between the original constituents of the composite [32], which follows some engineering procedures to increase the connectivity in-between the matrices and their reinforcements. Such an interphase may significantly influence various properties of the entire composite [33], even when it occupies a relatively small volume (or a small thickness) and this is especially true when the interphase area to its volume ratio is relatively high. Interestingly, this interphase may either increase the macroscopic stiffness, as for a bound rubber [34], or decrease it in the presence of interface defects and some other geometrical or material imperfections. 

The interface defects are considered in this work and the influence of uncertainty in their volume fraction in the given interphase on effective mechanical properties of some particulate composite is numerically studied using the generalized stochastic perturbation-based computational analysis. We verify the impact of this input uncertainty and, independently, of the strain level on the effective probabilistic stiffness tensor components, when particles are linear-elastic and the matrix is hyper-elastic. The iterative stochastic finite element method based on higher order Taylor expansions and polynomial bases is used to determine the first four probabilistic characteristics of the effective tensor components. Computational implementation has been completed using a hybrid application of the finite element method system Abaqus Standard and computer algebra system Maple 2017.

## 2. Theoretical Background

**Figure 1:**
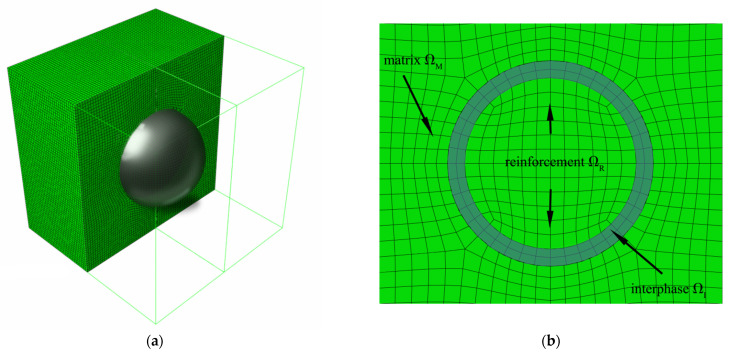
View (**a**) and cross-section (**b**) of the RVE.

Let us consider a periodic particulate composite Ω, whose particles are spherical and surrounded with an interphase ${\Omega =\Omega }_{R}{+\Omega }_{I}{+\Omega }_{M}$. The particles remain in an elastic isotropic reversible regime, while the matrix and the interphase are hyper-elastic and isotropic. The interphase is a constituent of the composite, whose properties are different (weaker) than those of the neighboring faces. The weakening effect is caused by the interface defects, specifically air voids located on the particle–matrix boundary. Each void is semi-spherical, is localized with its diameter on the particle surface and is directed outwards from the particle center. An interface defect is statistically scattered according to the Gaussian distribution radius $R_{\left(i,j\right)}$ and this radius is uniquely defined by its first two moments $E\left[R_{\left(i,j\right)}\right]$ and $\mathrm{Var}\left[R_{\left(i,j\right)}\right]$ at the given particle–matrix interface. The interface around any particle of this composite has a thickness of $\Delta_{j}=E\left[R_{\left(i,j\right)}\right]+3\sqrt{\mathrm{Var}\left[R_{\left(i,j\right)}\right]},\;j=1\;\ldots \;r$ [19,35]. The volume fraction of the interface defects w is calculated as a ratio of the volume of the interface defects and the volume of the interphase as $V_{D}{/V}_{I}$. The volume of defects is calculated as $V_{d}=n\cdot {2/3\Pi R}_{\left(i,j\right)}{}^{3}$, where n is the number of defects at the particle–matrix interface. All the phases of this composite are isotropic and its possible anisotropy may result from the accidental location of the particles into the larger composite volume only. Let us further assume that the reinforcing particle is significantly stiffer than the matrix, and a spatial distribution of the particles is periodic, while their volume is small in comparison to the entire composite specimen; this composite RVE is shown schematically in Figure 1a. The particles are so stiff that their deformation is relatively small, and we finally assume that these particles do not directly interact with each other, so that the initial symmetries in the internal microstructure hold true during the entire cyclic deformation process.

Next, the effective composite stiffness $C_{\mathrm{ij}}^{\mathrm{eff}}$ is introduced and with the aforementioned assumptions we consider only three components $C_{11}^{\mathrm{eff}}$, $C_{12}^{\mathrm{eff}}$ and $C_{44}^{\mathrm{eff}}$ independent from each other. All of them naturally depend upon the strain level applied on the RVE

(1) $$C_{\mathrm{kl}}^{\mathrm{eff}}\left(\varepsilon_{\mathrm{ij}}\right)\left[\begin{array}{cccccc} C_{11}^{\mathrm{eff}}\left(\varepsilon_{\mathrm{ij}}\right) & C_{12}^{\mathrm{eff}}\left(\varepsilon_{\mathrm{ij}}\right) & C_{12}^{\mathrm{eff}}\left(\varepsilon_{\mathrm{ij}}\right) & 0 & 0 & 0\\ & C_{11}^{\mathrm{eff}}\left(\varepsilon_{\mathrm{ij}}\right) & C_{12}^{\mathrm{eff}}\left(\varepsilon_{\mathrm{ij}}\right) & 0 & 0 & 0\\ & & C_{11}^{\mathrm{eff}}\left(\varepsilon_{\mathrm{ij}}\right) & 0 & 0 & 0\\ & & & C_{44}^{\mathrm{eff}}\left(\varepsilon_{\mathrm{ij}}\right) & 0 & 0\\ & \mathrm{sym}. & & & C_{44}^{\mathrm{eff}}\left(\varepsilon_{\mathrm{ij}}\right) & 0\\ & & & & & C_{44}^{\mathrm{eff}}\left(\varepsilon_{\mathrm{ij}}\right) \end{array}\right]$$

Energy contributions to this effective stiffness come from the hyper-elastic matrix and the interphase as well as from the elastic particles

(2) $${\int^{\;}}_{\Omega }C_{\alpha \beta \chi \delta }^{\left(\mathrm{eff}\right)}\varepsilon_{\alpha \beta }^{\prime }\varepsilon_{\chi \delta }^{\prime }d\Omega ={\int^{\;}}_{\Omega }C_{\alpha \beta \chi \delta }^{\left(R\right)}\varepsilon_{\alpha \beta }^{\prime }\varepsilon_{\chi \delta }^{\prime }+\;{\int^{\;}}_{\Omega }C_{\alpha \beta \chi \delta }^{\left(M\right)}\varepsilon_{\alpha \beta }^{\prime }\varepsilon_{\chi \delta }^{\prime }+\;{\int^{\;}}_{\Omega }C_{\alpha \beta \chi \delta }^{\left(I\right)}\varepsilon_{\alpha \beta }^{\prime }\varepsilon_{\chi \delta }^{\prime }\;d\Omega$$

The interphase and the matrix work according to the Arruda–Boyce hyper-elastic potential [36]. This potential could be read as

(3) $$U_{\mathrm{AB}}{=C}_{1}\left(\left(\frac{1}{2}I_{1}-3\right)+\frac{1}{{20\lambda }_{A}^{2}}+\frac{1}{{1050\lambda }_{A}^{4}}\left(I_{1}^{3}-27\right)+\frac{19}{{7000\lambda }_{A}^{6}}\left(I_{1}^{4}-81\right)+\frac{519}{{673750\lambda }_{A}^{8}}\left(I_{1}^{5}-243\right)\right)$$

where the two unknown parameters of the matrix $C_{1}$ and $\lambda_{A}$ are determined on the basis of the laboratory tests obtained for the high-density polyurethane (HDPU) Laripur LPR5020 reinforced with the carbon black (fullerenes C_60_). It was selected as the closest approximation of the matrix response coming from laboratory tests in this specific case study. A constitutive relation of the interphase $\sigma_{I,\mathrm{ij}}\left(\varepsilon_{\mathrm{ij}}\right)$ is considered as hyper-elastic according to the Arruda–Boyce theory. It is calculated from the rule of mixture, where the weakening of the entire stress–strain curve of the interphase in respect to the one of the matrices is inversely proportional to the volume fraction of defects $\sigma_{I,\mathrm{ij}}\left(\varepsilon_{\mathrm{ij}}\right){=\sigma }_{M,\mathrm{ij}}\left(\varepsilon_{\mathrm{ij}}\right)\cdot \left(1-w\right)$. It is adopted because the defects do not contribute to the stiffness of the interphase so that $\sigma_{D,\mathrm{ij}}\left(\varepsilon_{\mathrm{ij}}\right)=0$. Details of this interface model and its stochastic aspects can be found in [37]. Elastic properties of the particles include the Young modulus of $E_{R}=10\;\mathrm{GPa}$ and Poisson ratio of $\upsilon_{R\;}=0.3$. 

The representative volume element (RVE) of the composite used in the finite element method (FEM) computations has a single centrally located particle, which is surrounded by an interphase of constant thickness, and they are both inserted into the matrix; this composition is shown in Figure 1b. The carbon black particles (fullerenes C_60_) occupy 5% of the RVE, 5% of the interphase, and the last 90% of this composite is filled with a matrix. The mesh of this composite (Figure 1a) is made of about 50,000 20-noded brick finite elements with a second-order stress approximation; FEM computations are completed according to the implicit scheme using the full Newton technique including large strains.

A source of randomness in this work is the statistical dispersion of the volume fraction of interface defects w. Such a parameter is selected because the interface defects are extremely difficult to be directly measured and discretized, so that both their radius and total number at the particle–matrix interface vary for different particles and their interfaces. These two parameters both contribute to a dispersion of w, which is considered here as Gaussian parameter, having the following probability distribution function (PDF):

(4) $$p\left(w\right)=\frac{1}{\sigma \left(w\right)\sqrt{2\pi }}\exp\left(-\frac{{\left(w-E\left(w\right)\right)}^{2}}{{2\sigma }^{2}\left(w\right)}\right);\;w\in R$$

where E(w) is the expected value of the volume fraction of the interface defects and σ(w) is the standard deviation of this parameter. All further probabilistic calculations are conducted in the framework of the iterative stochastic finite element method (ISFEM) based on the generalized iterative stochastic perturbation technique [38]. In this method, the random input variable of $C_{\mathrm{ij}}^{\mathrm{eff}}$ is replaced by a Taylor series of nth order in the following way

(5) $$C_{\mathrm{ij}}^{\mathrm{eff}}\left({w,\varepsilon }_{\mathrm{ij}}\right){=C}_{\mathrm{ij}}^{\mathrm{eff},0}\left(w^{0}{,\varepsilon }_{\mathrm{ij}}\right)+\varepsilon {\frac{\partial C_{\mathrm{ij}}^{\mathrm{eff}}\left({w,\varepsilon }_{\mathrm{ij}}\right)}{\partial w}|}_{{w=w}^{0}}\Delta w+\ldots +\frac{\varepsilon^{n}}{n!}{\frac{\partial^{n}C_{\mathrm{ij}}^{\mathrm{eff}}\left({w,\varepsilon }_{\mathrm{ij}}\right)}{\partial w^{n}}|}_{{w=w}^{0}}\Delta w^{n}$$

ε in this equation is the so-called perturbation parameter, $C_{\mathrm{ij}}^{\mathrm{eff},0}\left(w^{0}{,\varepsilon }_{\mathrm{ij}}\right)$ constitutes the expected value of the input uncertain parameter, and the nth order variation is following $\varepsilon^{n}\Delta w^{n}$. The expected value of the uncertain parameter is calculated iteratively in the following manner:

(6) $$E\left(C_{\mathrm{ij}}^{\mathrm{eff}}\right){=C}_{\mathrm{ij}}^{\mathrm{eff},0}\left(w^{0}{,\varepsilon }_{\mathrm{ij}}\right)+\frac{\varepsilon^{2}}{2!}\frac{\partial^{2}C_{\mathrm{ij}}^{\mathrm{eff}}\left({w,\varepsilon }_{\mathrm{ij}}\right)}{\partial w^{2}}\mu_{2}\left({w,\varepsilon }_{\mathrm{ij}}\right)+\ldots +\frac{\varepsilon^{n}}{n!}\frac{\partial^{n}C_{\mathrm{ij}}^{\mathrm{eff}}\left({w,\varepsilon }_{\mathrm{ij}}\right)}{\partial w^{2}}\mu_{n}\left({w,\varepsilon }_{\mathrm{ij}}\right)$$

while the central moment for the Gaussian PDF $\mu_{p}$ is computed as

(7) $$\mu_{p}\left({w,\varepsilon }_{\mathrm{ij}}\right)=\{\begin{array}{c} 0;p=2k+1\\ \left(\sigma {\left({w,\varepsilon }_{\mathrm{ij}}\right)}^{p}\left(p-1\right)!!;\;p=2k\right) \end{array}$$

where p stands for the order of the given central probabilistic moment. The closed form formulas for higher order probabilistic characteristics in the presence of input Gaussian uncertainty are available in [38].

**Figure 2:**
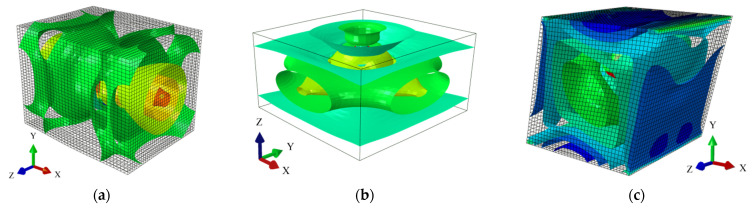
Principal strain isosurfaces for the (**a**) uniaxial and (**b**) biaxial tension, as well as (**c**) shear of the RVE.

Computations of the effective properties of the composite are made in the following order: firstly, a set of computations is completed in the FEM system Abaqus according to the boundary conditions shown schematically in Figure 2, together with the isosurfaces of the principal strain. They include prescribed uniaxial tensions, biaxial tensions and uniaxial shears that are applied on the RVE of the composite. The prescribed strains assigned on the outer edges are incrementally increased from the zero strain up to $\varepsilon_{\mathrm{ij}\;}=0.25$. This is performed for a set of volume fractions of defects w ∈ {0.1, 0.2…0.9} and in a ±10% neighborhood to these values of w. This effectively ends up in 98 nonlinear computations that are evenly placed in the domains of $\varepsilon_{\mathrm{ij}}\in \left(0,\;0.25\right)$ and w ∈ (0,0.9). The strain energies $U_{\mathrm{ij}}\left(\varepsilon_{\mathrm{ij}},w\right)$ coming from these stretches are then recalculated into effective stiffness $C_{\mathrm{ij}}^{\mathrm{eff}}$ according to the Arruda–Boyce hyper-elastic potential. These discrete results are approximated with use of the weighted least squares method (WLSM) with equal weights to retrieve a bivariate polynomial representation of $C_{\mathrm{ij}}^{\mathrm{eff}}\left(\varepsilon_{\mathrm{ij}},w\right)$, which is further called the response function. An order of polynomial is optimized with use of the minimization of the WLSM total error; such an approach proved to have an error below 5% for all data points taken into consideration. The effective stiffness is then subjected to symbolic procedures in the computer algebra program Maple 2017, which defines its first four probabilistic coefficients. In these computations an input uncertainty comes from the unknown coefficient of random dispersion of volume fraction of defects w. This uncertain parameter varies according to a Gaussian probability density function (PDF). Symbolic procedures are conducted according to the iterative stochastic finite element framework (ISFEM), where the probabilistic characteristics are defined independently, using a semi-analytical method (SAM), a crude Monte Carlo simulation (MCS) [39] with 50,000 trials per each realization, as well as using the stochastic perturbation technique with the statistically optimized order [38,40]. Characteristics include expected values, coefficients of variation, skewness and kurtosis, all as functions of the coefficient of random dispersion for the input random parameter (volume fraction of voids w). They are all based on the response function instead of the discrete data points of $C_{\mathrm{ij},k}^{\mathrm{eff}}$ coming directly from the FEM; this ensures their continuous character in the entire region of interest for $\left(\varepsilon_{\mathrm{ij}},w\right)$. This character, of course, does not hold for the MCS, which returns discrete results repeated to evenly cover the domain of interest. The SAM consists of numerical derivation of the probabilistic characteristics with a bound of ±3σ, where σ is the standard deviation of the input uncertain parameter w. Further numerical results are presented here separately for the three components of $C_{\mathrm{ij}}^{\mathrm{eff}}$ resulting from unique stretches of the RVE shown in Figure 2, i.e., for uniaxial tension, biaxial tension and uniaxial shear. 

## 3. Composite Simulation Results

**Figure 3:**
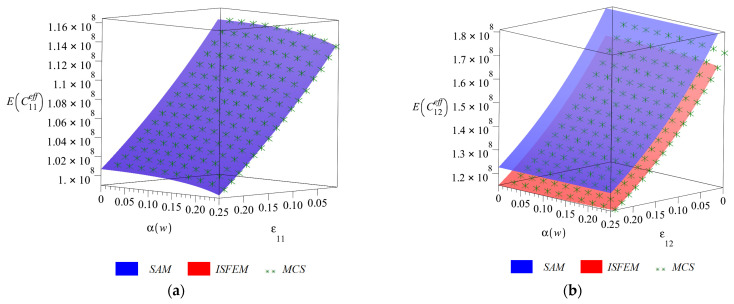

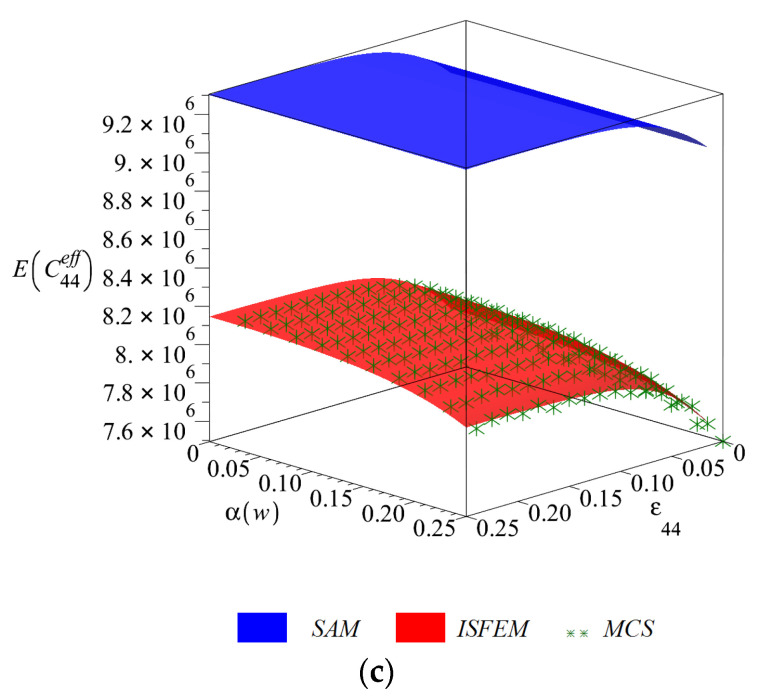
Expected values (**a**) $E\left(C_{11}^{\mathrm{eff}}\right)$, (**b**) $E\left(C_{12}^{\mathrm{eff}}\right)$ and (**c**) $E\left(C_{44}^{\mathrm{eff}}\right)$ w.r.t. α(w) and corresponding strain $\varepsilon_{\mathrm{ij}}$.

**Figure 4:**
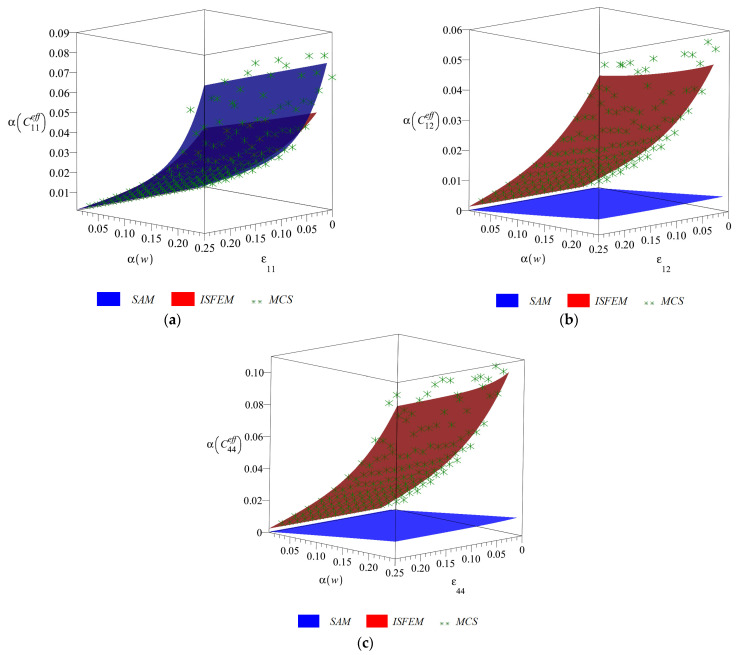
Coefficients of variation (**a**) $\alpha \left(C_{11}^{\mathrm{eff}}\right)$, (**b**) $\alpha \left(C_{12}^{\mathrm{eff}}\right)$ and (**c**) $\alpha \left(C_{44}^{\mathrm{eff}}\right)$ w.r.t. α(w) and corresponding strain $\varepsilon_{\mathrm{ij}}$.

**Figure 5:**
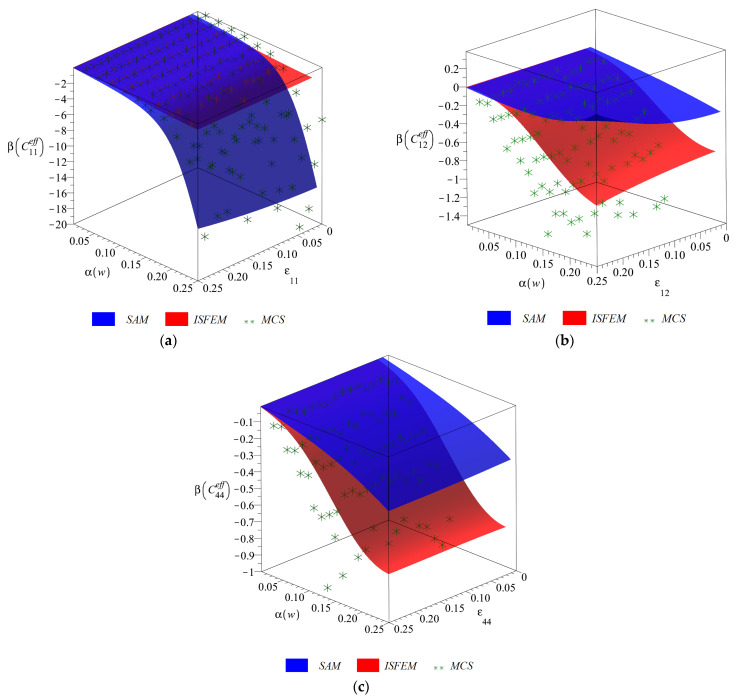
Skewness (**a**) $\beta \left(C_{11}^{\mathrm{eff}}\right)$, (**b**) $\beta \left(C_{12}^{\mathrm{eff}}\right)$ and (**c**) $\beta \left(C_{44}^{\mathrm{eff}}\right)$ w.r.t. α(w) and corresponding strain $\varepsilon_{\mathrm{ij}}$.

**Figure 6:**
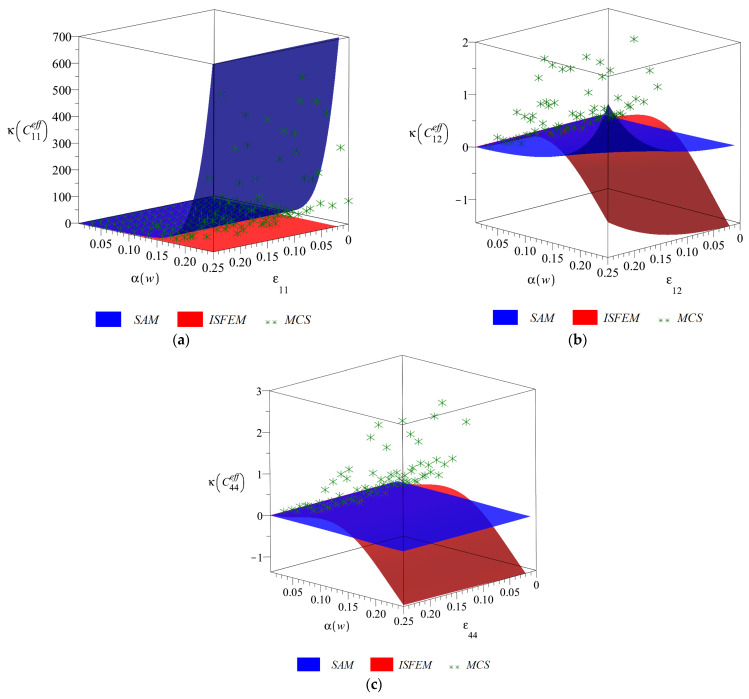
Kurtosis (**a**) $\kappa \left(C_{11}^{\mathrm{eff}}\right)$, (**b**) $\kappa \left(C_{12}^{\mathrm{eff}}\right)$ and (**c**) $\kappa \left(C_{44}^{\mathrm{eff}}\right)$ w.r.t. α(w) and corresponding strain $\varepsilon_{\mathrm{ij}}$.

Final diagrams of the effective stiffness tensor (Figure 3, Figure 4, Figure 5 and Figure 6) $C_{\mathrm{ij}}^{\mathrm{eff}}$ include its first four probabilistic coefficients, i.e., expected value $E\left(C_{\mathrm{ij}}^{\mathrm{eff}}\right)$, coefficient of variation $\alpha \left(C_{\mathrm{ij}}^{\mathrm{eff}}\right)$, skewness $\beta \left(C_{\mathrm{ij}}^{\mathrm{eff}}\right)$ and kurtosis $\kappa \left(C_{\mathrm{ij}}^{\mathrm{eff}}\right)$ in the domain of the input coefficient of the random dispersion of the volume fraction of defects α(w) ∈ (0, 0.25) and the corresponding strain $\varepsilon_{\mathrm{ij}}\in \left(0,\;0.25\right)$. Each of the three-dimensional graphs is presented for the three components of this effective stiffness ($C_{11}^{\mathrm{eff}}$, $C_{12}^{\mathrm{eff}}$ and $C_{44}^{\mathrm{eff}}$), and is computed for the expected value of volume fraction of defects of E(w)=0.05. The characteristics of the uniaxial coefficient are exclusively shown in part (a), the biaxial ones in part (b) and shearing ones in part (c) of these graphs. Colors on these graphs distinguish the three independent probabilistic methods applied, i.e., the iterative stochastic finite element method (ISFEM, red color), the Monte Carlo simulation (MCS, green color) and the semi-analytical method (SAM, blue color) that were used in the computations. The ISFEM and SAM produce continuous results in the entire domain of α(w) and $\varepsilon_{\mathrm{ij}}$, while the MCS discrete points are evenly distributed through this domain, which is a trait of the MCS. In the following paragraph, the general properties of the resulting probabilistic characteristics are reported and in the consecutive ones each characteristic is considered separately. 

The expected values and the coefficients of variation for all the components and probabilistic methods are exclusively positive, skewness is predominantly negative and kurtosis is positive for the MCS and SAM, while negative for the ISFEM. All the coefficients, despite the expected values, increase in magnitude together with an increase in input uncertainty α(w) and always have a smaller dispersion than the input parameter (volume fraction of defects) up to 3.5 times. This means that the stiffness is less spread than w. This is because defects occupy only a very limited volume of the composite $-\Omega_{d}=0.0025\;\Omega$. The resulting PDFs of the effective stiffness coefficients $C_{\mathrm{ij}}^{\mathrm{eff}}$ could not be Gaussian because of the nonzero skewness and kurtoses for practically the entire domain of interest in α(w) and $\varepsilon_{\mathrm{ij}}$. Skew for these PDFs is negative and the concentration of probability density close to the expected value is relatively high. An influence of the strain $\varepsilon_{\mathrm{ij}}$ is mostly visible for $E\left(C_{\mathrm{ij}}^{\mathrm{eff}}\right)$ and it is not high for $\alpha \left(C_{\mathrm{ij}}^{\mathrm{eff}}\right)$, $\beta \left(C_{\mathrm{ij}}^{\mathrm{eff}}\right)$ and $\kappa \left(C_{\mathrm{ij}}^{\mathrm{eff}}\right)$, where it is still not negligible. Unlike for the stiffness tensor in linear elastic materials, the three probabilistic methods are not perfectly matching for the expected values and coefficients of variation. While the MCS and the ISFEM return exactly the same results for $E\left(C_{\mathrm{ij}}^{\mathrm{eff}}\right)$ and $\alpha \left(C_{\mathrm{ij}}^{\mathrm{eff}}\right)$, the SAM is a little distant. When skewness and kurtosis is considered, either the ISFEM returns the results between the MCS and SAM or the MCS is very close to the SAM. Differences in-between the three probabilistic methods generally increase together with an increase in the order of characteristics; this is especially visible for kurtosis. All the probabilistic characteristics differ in magnitude and dependence on $\varepsilon_{\mathrm{ij}}$ and α(w) for the respective stiffness coefficients and, because of this, application of a single PDF for all the stiffness coefficients would be erroneous for this specific material. 

The expected values of the effective stiffness tensor $E\left(C_{\mathrm{ij}}^{\mathrm{eff}}\right)$ are presented in Figure 3 in function of α(w) and $\varepsilon_{\mathrm{ij}}$. The ones corresponding to uniaxial tension (Figure 3a) and biaxial tension (Figure 3b) are decreasing with an increase in strain, while the shearing one (Figure 3c) slightly increases by up to 4%. The steepest rate of change together with $\varepsilon_{\mathrm{ij}}$ has $E\left(C_{12}^{\mathrm{eff}}\right)$ at around 1.6% per percent of strain and the other components below 1% per percent of strain. An increase in the scatter of the input uncertain parameter α(w) does not at all affect $E\left(C_{12}^{\mathrm{eff}}\right)$, while it causes a decrease in $E\left(C_{11}^{\mathrm{eff}}\right)$ and $E\left(C_{44}^{\mathrm{eff}}\right)$ of less than 1%. The three probabilistic methods return perfectly the same results for the uniaxial coefficient, while the SAM overestimates the expectation around 1% for the biaxial and up to 10% for the shearing component.

The coefficients of variation of the effective stiffness tensor $\alpha \left(C_{\mathrm{ij}}^{\mathrm{eff}}\right)$ are reported in Figure 4. They always increase in an exponential manner together with an increase in α(w), are much less affected by $\varepsilon_{\mathrm{ij}}$ than by α(w) and are always lower than the coefficient of random dispersion of the input (α(w)). The components corresponding to tension slightly increase together with an increase in $\varepsilon_{\mathrm{ij}}$, while the one corresponding to shear decreases. This change of magnitude, however, does not exceed 5%. The ISFEM and MCS return perfectly the same results and SAM always underestimates the $\alpha \left(C_{\mathrm{ij}}^{\mathrm{eff}}\right)$; only for $C_{11}^{\mathrm{eff}}$ are the results of SAM close to the other methods, especially for α(w) < 0.15. The coefficient of variation is a little higher for the shear component (maximum of 0.1) than for the tensional ones, where it reaches up to 0.07. Obviously, for α(w)=0 the $\alpha \left(C_{\mathrm{ij}}^{\mathrm{eff}}\right)$ also approaches zero. 

Skewness of the effective stiffness $\beta \left(C_{\mathrm{ij}}^{\mathrm{eff}}\right)$ is presented in Figure 5. It is always negative for all the stiffness components, approaches zero for α(w)=0 and has considerable differences in magnitude for each of $C_{\mathrm{ij}}^{\mathrm{eff}}$. It either increases ($\beta \left(C_{12}^{\mathrm{eff}}\right)$ and $\beta \left(C_{44}^{\mathrm{eff}}\right)$) or decreases ($\beta \left(C_{11}^{\mathrm{eff}}\right)$) together with an increase in $\varepsilon_{\mathrm{ij}}$. Similarly to the coefficient of variation, these changes are relatively small and below 5%. Results reported by the three probabilistic methods slightly differ from each other. The higher bound of skewness is always returned by the MCS, the lower one by the SAM and the ISFEM returns results in-between the other methods, close to the MCS. This is not true for $\beta \left(C_{11}^{\mathrm{eff}}\right)$, where the ISFEM is distant and smaller from the other methods. For this component the ISFEM reports skewness of magnitude close to other stiffness components ($\min\left(\beta \left(C_{44}^{\mathrm{eff}}\right)\right)=-2$ $\min\left(\beta \left(C_{44}^{\mathrm{eff}}\right)\right)=-0.9$ and $\min\left(\beta \left(C_{44}^{\mathrm{eff}}\right)\right)=-1.4$) and the other methods seem to overestimate it.

Kurtosis of all the effective stiffness tensor components is presented in Figure 6 in relation to α(w) and $\varepsilon_{\mathrm{ij}}$. It is either only positive ($\kappa \left(C_{11}^{\mathrm{eff}}\right)$), or positive for the SAM and MCS and negative for the ISFEM ($\kappa \left(C_{12}^{\mathrm{eff}}\right)$ and $\kappa \left(C_{44}^{\mathrm{eff}}\right)$). The agreement of the three probabilistic methods is not perfect and the weakest from all of the considered characteristics. It is the ISFEM that is the most distant especially for α(w) > 0.1 and produces always the smallest result; it underestimates the kurtosis. The influence of $\varepsilon_{\mathrm{ij}}$ is especially relevant for $\kappa \left(C_{12}^{\mathrm{eff}}\right)$, where all three methods return an increase in $\kappa \left(C_{\mathrm{ij}}^{\mathrm{eff}}\right)$ together with an increase in $\varepsilon_{\mathrm{ij}}.$ Kurtosis is monotonic with respect to $\varepsilon_{\mathrm{ij}}$ and α(w), it always increases magnitude together with an increase in α(w) and converges to zero as α(w)=0.

## 4. Concluding Remarks

This work reports a successful determination of the random nonlinear cubic effective stiffness tensor $C_{\mathrm{ij}}^{\mathrm{eff}}$ of particulate composites with spherical reinforcement and with an interface with stochastic imperfections and it is calculated in the framework of the iterative stochastic finite element method. Probabilistic characteristics are determined using three concurrent numerical methods: the semi-analytical method, Monte Carlo simulation, and stochastic perturbation method. This study concerns a composite with HDPU matrix reinforced with C_60_ spherical fullerenes surrounded by the interphase defects. It is conducted in a hyper-elastic regime of this material, where the constitutive relation of the matrix has been adopted after initial laboratory tests. 

The resulting effective stiffness tensor is sensitive to uncertainty in the volume fraction of the interface defects and it cannot have a Gaussian distribution regardless of the strain intensity applied. It has more than twice the lower uncertainty level than the input one and, unlike for the linear elastic case, its probabilistic characteristics highly depend upon the strain imposed on the RVE; this dependence has no evident pattern for the respective components and different probabilistic characteristics but it is basically monotonic. An influence of statistical scattering of the interface defects on the expected values of the effective stiffness tensor components is relatively small (limited to ±1%) but it is remarkably larger when higher order statistics of this tensor are taken into account.

A coincidence of the three probabilistic methods is relatively high for the expectations $E\left(C_{\mathrm{ij}}^{\mathrm{eff}}\right)$ and coefficients of variation $\alpha \left(C_{\mathrm{ij}}^{\mathrm{eff}}\right)$, where the ISFEM and MCS perfectly match with each other and are limited for the higher order characteristics, such as skewness $\beta \left(C_{\mathrm{ij}}^{\mathrm{eff}}\right)$ and kurtosis $\kappa \left(C_{\mathrm{ij}}^{\mathrm{eff}}\right)$. The ISFEM usually returns result in-between the MCS (higher bound) and SAM (lower bound) for $\beta \left(C_{\mathrm{ij}}^{\mathrm{eff}}\right)$, while it is in agreement with the other methods in the case of $\kappa \left(C_{\mathrm{ij}}^{\mathrm{eff}}\right)$ when α(w) < 0.08. 

There is no doubt that experimentally-based homogenization of particulate composites with stochastic interface defects brings new interesting results, so that further SFEM computational studies will concern numerical simulation of hysteresis of such a composite in the probabilistic context. 

## Data Availability

Not applicable.
